# Progressive multifocal leukoencephalopathy in a patient without apparent immunosuppression

**DOI:** 10.1186/1743-422X-7-256

**Published:** 2010-09-28

**Authors:** Christos Vaklavas, Elsa P Sotelo-Rafiq, Jordan Lovy, Miguel A Escobar, Apostolia M Tsimberidou

**Affiliations:** 1 Department of Internal Medicine, The University of Texas Medical School at Houston, Houston, Texas, USA; 2 Department of Pathology and Laboratory Medicine, The University of Texas Medical School at Houston, Houston, Texas, USA; 3 IPC Houston, Hospitalist Co, Houston, Texas, USA; 4 Department of Investigational Cancer Therapeutics, The University of Texas M. D. Anderson Cancer Center, Houston, Texas, USA

## Abstract

An 80-year-old man with no history of an immune-compromising disorder was diagnosed with progressive multifocal leukoencephalopathy (PML). He presented with dysphagia and left-sided weakness; magnetic resonance imaging demonstrated marked signal abnormality in the subcortical white matter of the left frontal lobe and in the posterior limb of the right internal capsule. Polymerase chain reaction (PCR) analysis of the cerebrospinal fluid (CSF) was negative for John Cunningham (JC) virus. On brain biopsy, foamy macrophages infiltrating the white matter were identified, staining positive for anti-simian virus 40 antibodies. Postoperatively, PCR for JC viral DNA in the CSF was positive, establishing the diagnosis of PML. Extensive investigation for an occult immunocompromising disorder was negative. The patient's neurologic deficits rapidly increased throughout his hospital stay, and he died 3.5 months after his diagnosis.

## Introduction

Progressive multifocal leukoencephalopathy (PML) is a rapidly advancing demyelinating disorder of the central nervous system almost exclusively encountered in immunocompromised individuals[1]. It is caused by reactivation of the John Cunningham virus (JCV) under conditions of cellular immunocompromise such as those encountered in patients with acquired immunodeficiency syndrome (AIDS), patients with hematologic and solid organ malignancies receiving chemotherapy, and transplant recipients under immunosuppression[2]. The interest in this disease has recently increased because of its association with natalizumab, a monoclonal antibody directed against α_4 _integrins that is used to treat Crohn's disease[3] and multiple sclerosis[4].

Here, we describe a rare occurrence of PML, with a rapidly fatal outcome, in a patient without an apparent history of immunosuppression.

## Case Report

An 80-year-old white man with a medical history of hypertension presented to the emergency department of an outside hospital after sustaining a fall. He had begun to drag his left leg at least one month prior to presentation, and over the preceding 5 days he had started to use a walker. The left-sided weakness was accompanied by progressive dysphagia, which occurred initially with fast eating and later with a regular eating rate. He had lost 35 lbs over the preceding 3 months. He had visited the emergency center monthly in the preceding 3 months because of falls. The patient was started on aspirin 25 mg/extended-release dipyridamole (Aggrenox^®^) 200 mg PO daily for suspected transient ischemic attacks.

At the time of presentation at Tyler County Hospital on May 16, 2008, a computed tomography (CT) of the patient's head demonstrated multiple cerebrovascular accidents. Cerebral atrophy and old infarcts in the right internal capsule, right cerebral peduncle, right thalamus, and left frontal lobe were identified. The patient was transferred from Tyler County Hospital in Woodville, Texas, to Christus St. Elizabeth Hospital in Beaumont, Texas, for a higher level of care.

Upon physical examination by the physicians at Christus St. Elizabeth Hospital, the patient's blood pressure was 158/81 mmHg, his respiratory rate 12 breaths per minute, heart rate 90 beats per minute, and temperature 37°Celsius. The patient was described as a cachectic Caucasian man, alert, awake, and oriented to person, time, and place. His speech was fluent; comprehension, naming, and repetition were intact. Examination of the cranial nerves showed mild drooping of the left angle of the mouth; all other cranial nerves were intact. Left hemiparesis was noted; motor strength was 4+/5 on the left symmetrically in the upper and lower extremities and 5-/5 in the right extremities. Deep tendon reflexes were hyperactive, but no extensor plantar responses were recorded. On admission to Christus St. Elizabeth Hospital, complete blood count and chemistry levels were normal, except for blood urea nitrogen and serum creatinine levels of 37 mg/dL and 2.4 mg/dL, respectively, indicating acute or chronic nephropathy. There was no history of renal insufficiency.

On his second hospital day, a magnetic resonance imaging (MRI) study of the brain without contrast demonstrated ill-defined white matter edema involving the right thalamus and left paramidline frontal lobe (sparing the cortex) and extending into the right cerebral peduncle. The lesions appeared bright on fluid-attenuated inversion recovery (FLAIR) sequences and diffusion images.

The findings raised concern about an underlying malignancy. Clinical findings and imaging studies (CT of the chest/abdomen/pelvis) did not, however, demonstrate any suspicious lesions. Immunofixation analysis was negative for antineuronal nuclear antibodies type 1 (anti-Hu) and 2 (anti-Ri). Renal and transrectal ultrasonography were conducted on the third inpatient day, prompted by the patient's newly diagnosed renal insufficiency, without contributory findings. A spinal tap yielded clear, colorless cerebrospinal fluid (CSF) with the following component levels: glucose 55 mg/dL (serum level, 88 mg/dL) and protein 31 mg/dL. Gram staining and culture were negative. On cytospin preparations, 10 cells were collected: 70% normal lymphocytes and 30% monocytes. A specimen was sent for polymerase chain reaction (PCR) analysis for JC virus and herpes simplex virus; both studies were negative. The CSF was also negative for the presence of *Borrelia burgdorferi *antigens. Myelin basic protein was elevated (12.63 ng/mL, reference < 4 ng/mL); no oligoclonal bands were identified. An electroencephalogram demonstrated mild bifrontal slowing, more prominent on the left than the right. A carotid Doppler ultrasound was normal. Serum and urine protein electrophoreses were consistent with proteinuria of glomerular pattern but negative for the presence of paraprotein.

On day 11, the patient experienced deterioration of his neurologic symptoms. On neurologic examination, the patient was awake but drowsy and oriented to time, place, and person. Mild left facial nerve palsy was again noted. Motor strength was 5/5 on the right, whereas on the left there was progressive weakness; motor strength was 2/5 on the left hand, 4-/5 on the left biceps, and 4-/5 over the left leg. Deep tendon reflexes were 2+, and no extensor plantar responses were reported. Coordination was normal on the right side, whereas some ataxia was recorded on the left. MRI without contrast of the cervical, thoracic, and lumbar spine on day 12 demonstrated no major stenosis or neural impingement. MRI of the brain on day 13 demonstrated two areas of abnormal signals on diffusion-weighted imaging (left frontal lobe measuring 2.7 × 2.7 cm and right thalamus and basal ganglion measuring 1.6 × 1.2 cm). FLAIR images were positive in these areas and in several other small areas elsewhere. No significant change was identified when this study was compared with the one obtained on the second inpatient day.

A brain biopsy was considered, and the patient was transferred to Memorial Hermann Hospital, Houston, Texas, on June 6, 2008. On admission, his vital signs were within normal limits. He was described as a cachectic Caucasian man with a poor performance status. His neurologic examination was remarkable for left facial drooping and left-sided weakness (1/5 in the left upper and 2/5 in the left lower extremity). Deep tendon reflexes were hyperactive on the left side and normal on the right side. In addition, pinprick, hot and cold, light touch, and vibratory sensation and proprioception were decreased on the left side. Gait could not be assessed due to his left motor weakness. His Folstein Mini Mental State Examination score was 23/30; orientation was intact, whereas registration (1/3) and recollection (0/3) were severely affected.

**Figure 1:**
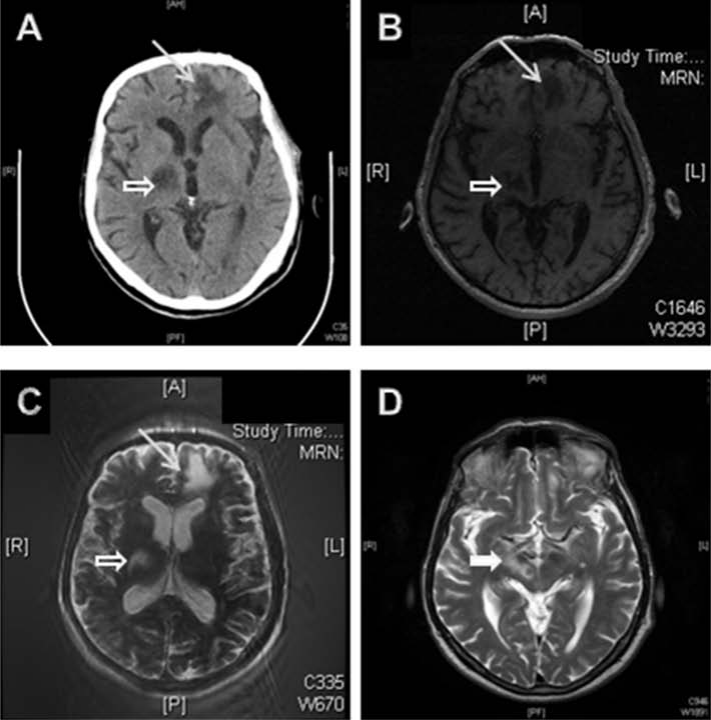
**Imaging studies conducted in the final hospital**. **A**, computed tomography of the head without contrast. **B**, T1-weighted magnetic resonance imaging of the head. **C **and **D**, T2-weighted magnetic resonance imaging of the head. The arrows indicate the subcortical lesion in the left frontal lobe; the block arrows indicate the lesion in the right posterior limb of the internal capsule and thalamus extending along the corticospinal tract (solid arrow, image D)

A repeat MRI at Memorial Hermann Hospital showed two prominent areas of signal abnormality: one in the subcortical white matter of the left frontal lobe and one in the right posterior limb of the internal capsule and thalamus extending along the corticospinal tract as far down as the pons. The lesions were hypointense on T1-weighted images and hyperintense on T2-weighted images. The cortex was spared (figure 1).

**Figure 2:**
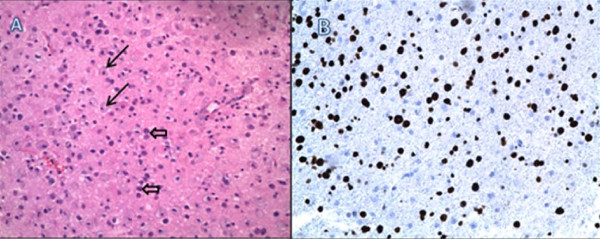
**Biopsy specimen of the brain lesion. A**, infiltrating foamy macrophages (arrows) and reactive astrocytes (block arrows) on hematoxylin and eosin stain of a deep white matter biopsy. **B**, positive stain for simian virus 40 (stains polyomavirus in humans).

Twenty-five days after his initial presentation, the patient underwent image-guided open brain biopsy through a left frontal craniotomy. Hematoxylin and eosin staining of the biopsy tissue revealed foamy macrophages infiltrating the brain tissue. Reactive gliosis with bizarre astrocytes was also seen. Stains for herpes simplex virus, cytomegalovirus, adenovirus, and *Toxoplasma gondii *were negative. Immunohistochemical staining with anti-simian virus 40 antibody was positive. The latter antibody stains positive for human polyomaviruses (JC virus, BK virus; KI polyomavirus, WU polyomavirus, and Merkel cell polyomavirus[5]) (figure 2). PCR of the CSF for JCV conducted in the same laboratory as previously was positive, establishing the diagnosis. The sensitivity level of the PCR was not available in the report but generally the sensitivity is 10^-3 ^to 10^-4^.

This unexpected finding spawned a thorough investigation for an occult immunocompromising disorder. Testing for the human immunodeficiency virus (HIV) was negative by enzyme immunoassay (twice) and PCR. The patient's CD4 count was 332 cells/μl (normal range, 410-1590 cells/μl) and his CD8 count was 485 cells/μl (normal range, 190-1140 cells/μl), as measured by flow cytometry. Testing for other infectious causes (toxoplasmosis, hepatitides, *Cryptococcus*, human T-lymphotropic virus I and II) was also negative. His persistent lymphopenia (absolute lymphocyte count throughout the hospital course ranged between 120 and 1077 cells/μl) raised the suspicion for a rheumatologic disease. Athough his antinuclear antibody titer was high (1:1280), he did not meet any clinical criteria for lupus erythematosus. Testing for anti-Ro and anti-La autoantibodies was positive, but the absence of xerostomia/xerophthalmia and a negative biopsy of minor salivary glands did not substantiate the diagnosis of Sjögren syndrome. The rest of the rheumatologic testing (anti-double-stranded DNA, cyclic citrullinated peptide, anti-smooth muscle and anti-RNP antibodies; rapid plasma reagin; complement 3 and 4 levels) was unrevealing. A bone marrow biopsy revealed variable cellularity (10-30%), with trilineage hematopoiesis and increased iron stores. There was no morphologic evidence of malignancy. Stains and cultures for acid-fast bacilli and fungi and cultures for cytomegalovirus were negative. The chromosomal analysis was negative as well.

Throughout the rest of his hospital stay, the patient's neurologic condition continued to deteriorate. His course was marked by an episode of altered mental status, progressive dementia, motor weakness, declining visual acuity, and worsening performance status. He was discharged to a skilled nursing facility closer to home, where he died 3.5 months after the diagnosis of PML.

## Discussion

Progressive multifocal leukoencephalopathy was originally described in 1958 in two patients with chronic lymphocytic leukemia and one patient with Hodgkin's disease[6]. The causative agent, the JC virus, was isolated in 1971 from the brain of a patient with Hodgkin's disease, and the virus was named after him[7]. With the advent of the HIV epidemic, PML was recognized as a major opportunistic infection of AIDS,[8] but with effective antiretroviral therapy, its incidence and attributable mortality rates have decreased[9]. Most recently, interest in PML has been revived by its association with natalizumab (Tysabri^®^), a promising new drug for the treatment of multiple sclerosis and Crohn's disease[2].

The occurrence of PML in persons without known immunosuppression is exceedingly rare. Although traditionally associated with conditions of cellular immunocompromise, the concept that profound cellular immunosuppression is required for the reactivation of the JC virus has recently been challenged [10]. Although old age has been associated with idiopathic CD4+ lymphocytopenia,[11] and the latter has been associated with PML,[10,12] our patient did not meet the Centers for Disease Control and Prevention criteria for idiopathic CD4+ lymphocytopenia [13]. With his lymphocyte count ranging between 120 and 1077 lymphocytes per microliter, however, it is possible that he had transient CD4+ lymphocytopenia. The association of aging with CD4+ lymphocytopenia[11] and the high JCV seroprevalence in adults[14] should increase awareness about the possible diagnosis of PML in the appropriate clinical and radiographic setting.

The initially negative PCR results for JC virus in the CSF, despite the suspicious radiologic findings, delayed the diagnosis. Negative PCR results for JCV-DNA in the CSF have been described in patients with AIDS,[15] and a correlation with active antiretroviral treatment has been hypothesized[1]. CSF obtained after open brain biopsy was positive for JCV-DNA, indicating that a breach in the blood-brain barrier or progressive infection may have led to the dissemination of the virus in the subarachnoid space. The fact that a negative PCR result for JCV-DNA cannot completely exclude PML has raised the need for a new consensus terminology, in which immunosuppressed patients with clinical and radiographic features consistent with PML and no alternative etiology should be considered as "possible PML"[1].

The occurrence of PML in patients without identifiable immunodeficiency poses a challenge from a therapeutic perspective. While strategies to reverse immunodeficiency, such as discontinuation of immunosuppressive therapy, institution of antiretroviral therapy in HIV-positive patients[1], plasma exchange and immunoadsorption in natalizumab treated patients [16], work well in certain groups, no treatment options of proven efficacy exist for patients without a clear immune disorder[17]. Cytarabine[18], cidofovir[19], topotecan[20], and mirtazapine[21] have been investigated as therapeutic agents, mostly in patients with AIDS, with variable results and toxicities. A study to investigate the effects of mefloquine in PML is currently ongoing[22].

## Consent

A consent has been waived by the Institutional Review Board for the publication of this case report.

## Competing interests

The authors declare that they have no competing interests.

## Authors' contributions

CV participated in the care of the patient and drafted the manuscript, EPSR examined the pathologic specimens and provided the pathologic figures, JL and MAE participated in the care of the patient, and AMT drafted the manuscript. All authors read and approved the final manuscript.
